# Associations of thyroid hormones with metabolic syndrome and coronary artery stenosis in euthyroid individuals: A cross-sectional study

**DOI:** 10.1097/MD.0000000000048975

**Published:** 2026-05-29

**Authors:** Mengran Guo, Chao Liu, Dan Lv, Qingshu Lin, Ali Zhang, Peng Cai

**Affiliations:** a Department of Critical Care Medicine, PLA 80th Group Army Hospital, Weifang, China; b Department of Emergency Medicine, Jinling Hospital, Affiliated Hospital of Medical School, Nanjing University, Nanjing, China; c Department of Cardiology, Institute of Field Surgery, Daping Hospital, Army Military Medical University, Chongqing, China.

**Keywords:** coronary artery stenosis, free triiodothyronine, Gensini score, metabolic syndrome, thyroid hormones

## Abstract

This study aimed to investigate the associations between thyroid hormones, metabolic syndrome (MetS), and coronary artery stenosis (CAS) in euthyroid patients, given the insufficient evidence on concurrent evaluation of these endpoints. In this cross-sectional study, demographic data, biochemical parameters, and coronary angiography were collected. The Gensini score was quantified to determine CAS severity. Participants were assigned to non-MetS and MetS groups, and further categorized into non-CAS, CAS I (stenosis < 50%), and CAS II (stenosis ≥ 50%) subgroups. The association between thyroid hormones, MetS, and CAS was analyzed. This study was approved by the Ethics Committee of the Army Medical Center of PLA. A total of 947 euthyroid adults were enrolled in the present study. Notably, free triiodothyronine (FT3) and the FT3/free thyroxine (FT4) ratio were significantly higher in the MetS group compared with the non-MetS group (all *P* < .05). In contrast, FT3, total triiodothyronine, and the FT3/FT4 ratio were markedly lower in the CAS II group than in the non-CAS group (all *P* < .05). Spearman correlation analysis in CAS patients showed that FT3, total triiodothyronine, and the FT3/FT4 ratio were negatively correlated with the Gensini score, and positively correlated with diastolic blood pressure, body mass index, and triglycerides (*P* < .05). After adjustment for potential confounders, multivariate linear regression analysis showed that FT3 remained significantly and linearly negatively correlated with the Gensini score (*P* < .001). In euthyroid individuals, thyroid hormones, particularly FT3, have inconsistent correlations with MetS and CAS, reflecting the complexity of thyroid hormone physiology.

## 1. Introduction

In recent years, there has been growing interest in the relationship between thyroid hormone levels and both metabolic syndrome (MetS) as well as coronary artery disease (CAD) in individuals with normal thyroid function.^[1]^ MetS is a well-established risk factor for CAD, as demonstrated by several large-scale clinical studies.^[2]^ Indicators, including blood glucose, triglycerides (TGs), and blood pressure, are positively associated with CAD.^[3]^ However, free triiodothyronine (FT3), the main active form of thyroid hormone, exhibits inconsistent associations. While some studies report a positive correlation between FT3 and the incidence of MetS, others suggest an inverse relationship between FT3 and coronary artery injury.^[4,5]^ Therefore, this apparent paradox warrants further investigation. FT3, the primary active form of thyroid hormone, plays a key role in regulating basal metabolic rate and also influences cardiovascular function by affecting lipid metabolism, vascular endothelial function, and sympathetic nervous system activity.^[4]^ Several cross-sectional studies have shown a positive association between FT3 levels and metabolic abnormalities.^[6]^ A prospective cohort study by Gu et al identified elevated FT3 levels as an independent predictor of MetS, potentially due to FT3’s role in promoting lipid breakdown and increasing insulin resistance.^[7]^ Previous studies found that FT3 levels were significantly higher in patients with sustained hypertension, suggesting that thyroid hormones may influence blood pressure regulation by enhancing sympathetic nerve activity. These findings are consistent with the well-established metabolic effects of thyroid hormones. Given the observed positive correlation between FT3 and MetS, along with the known association between metabolic indices and atherosclerosis, it is hypothesized that FT3 is also positively correlated with coronary atherosclerosis.

Nevertheless, the investigation of FT3 and CAD in individuals with normal thyroid function has revealed a contrasting trend. Shen et al analyzed data from 4221 patients who underwent coronary computed tomography angiography and found a significant negative correlation between FT3 levels and coronary artery calcification scores. Moreover, individuals in the highest FT3 quartile had a 37% lower risk of coronary artery calcification.^[8]^ Similarly, a study by He et al involving patients with diabetes mellitus and a history of myocardial infarction found that a low FT3 to free thyroxine (FT4) ratio was associated with a 2.5-fold increase in the risk of major cardiovascular events.^[9]^ These findings add to the ambiguity surrounding the true relationship between thyroid hormone levels, MetS, and CAD.

Current clinical research primarily focuses on isolated endpoints, such as MetS or coronary stenosis, and is further limited by substantial population heterogeneity and a lack of data derived from a single cohort. To address these limitations, we designed this study to perform coronary angiography and biochemical assessments within the same study population, enabling a more rigorous and precise evaluation of the relationship between thyroid hormone levels, metabolic parameters, and the extent of coronary stenosis.

## 2. Methods

### 2.1. Study design

The present cross-sectional study involved collecting demographic data, biochemical parameters, and coronary angiography findings. The Gensini score was used to determine coronary artery stenosis (CAS) severity. Participants were classified into non-MetS and MetS groups, and further categorized into non-CAS, CAS I, and CAS II subgroups according to coronary angiography outcomes. Relationships among thyroid hormones, MetS, and CAS were then analyzed. This study was approved by the Ethics Committee of the Army Medical Center of PLA (Ratification No:2019(126)). All participants provided written informed consent prior to their participation in this study. Moreover, our study was conducted in accordance with the Declaration of Helsinki.

### 2.2. Study participants

From December 2017 to March 2025, we randomly enrolled 2841 patients from the Department of Cardiovascular Medicine at the hospital. All participants presented with chest tightness or chest pain. Random sampling was performed using computer-generated random numbers. All eligible subjects were assigned unique identifiers, and random numbers were generated using statistical software for participant selection. The following individuals were excluded based on our exclusion criteria: 276 patients with abnormal thyroid function; 1376 patients using antihypertensive drugs, lipid-lowering agents, or medications affecting thyroid function; 32 patients diagnosed with congenital heart disease, cardiomyopathy, or moderate to severe valvular heart disease; 137 patients with a history of coronary artery bypass grafting or coronary stent implantation; and 73 patients with renal or hepatic insufficiency. After rigorous screening, 947 patients were included in the final analysis.

### 2.3. Participant grouping

Selective coronary angiography was performed in all patients via transradial arterial access. Based on the degree of coronary stenosis, patients showing no significant stenosis on coronary angiography were classified into the noncoronary stenosis group (non-CAS group). Those with <50% stenosis in the major coronary artery branches were placed in the mild CAS group (CAS I group). Patients with stenosis of 50% or greater in the principal coronary branches were assigned to the severe CAS group (CAS II group).^[10,11]^ Following coronary angiography, the extent of coronary stenosis was quantified using the Gensini scoring system. Lumen narrowing was categorized as 1% to 25%, 26% to 50%, 51% to 75%, 76% to 90%, 91% to 99%, and 100%, corresponding to scores of 1, 2, 4, 8, 16, and 32, respectively. Next, each stenosis score was multiplied by a coefficient based on the lesion’s location. The coefficient was 5 for lesions in the left main artery, 2.5 for lesions in the proximal segments of the left anterior descending and circumflex arteries, and 1.5 for lesions in the middle segment of the left anterior descending artery. Meanwhile, lesions in the distal segment of the left anterior descending artery, the first diagonal branch, the middle and distal segments of the circumflex artery, the obtuse marginal branch, the right coronary artery, and the posterior descending branch of the right coronary artery were assigned a coefficient of 1. In addition, lesions in the second diagonal branch, the posterior collateral branch of the right coronary artery, and other smaller branches were assigned a coefficient of 0.5. The overall severity of coronary stenosis for each patient was calculated as the sum of all individual vascular lesion scores.^[12]^ MetS was defined according to the diagnostic criteria established by the Diabetes Branch of the Chinese Medical Association, which include: overweight and/or obesity, defined as a body mass index (BMI) of 25 or greater; fasting blood glucose levels of 6.1 mmol/L (110 mg/dL) or higher, and/or 2-hour postprandial glucose levels of 7.8 mmol/L (140 mg/dL) or higher, and/or a prior diagnosis of diabetes with ongoing treatment; systolic blood pressure (SBP) and/or diastolic blood pressure (DBP) of 140/90 mm Hg or higher, and/or a diagnosis of hypertension requiring treatment; and fasting TG levels of 1.7 mmol/L (150 mg/dL) or higher, and/or fasting high-density lipoprotein (HDL) cholesterol levels below 0.9 mmol/L (35 mg/dL) in males or below 1.0 mmol/L (39 mg/dL) in females.^[13]^ A diagnosis of MetS is made when 3 or more of these criteria are met. Based on these standards, participants were further divided into the MetS group and the non-MetS group.

### 2.4. General data

Age, gender, smoking history, and drinking history were collected through questionnaires. Height and weight were measured on site, and BMI was calculated using the formula: BMI = weight (kg)/height^2^ (m^2^).^[14]^

### 2.5. Blood pressure measurement

After a 10-minute rest in the seated position, a qualified medical professional measured the blood pressure of the right upper arm’s brachial artery using a mercury sphygmomanometer. The final blood pressure value was calculated as the mean of 3 measurements obtained on separate days, each taken at least 12 hours apart.^[15]^

### 2.6. Biochemical detection

After a 12-hour fasting period, blood samples were collected from the anterior cubital vein between 6:00 am and 8:00 am. The concentrations of thyroid-stimulating hormone (TSH), total triiodothyronine (T3), total tetraiodothyronine (T4), FT3, and FT4 were measured using electrochemiluminescence on a Beckman DXI800 automated biochemical analyzer (Brea Company). TG, HDL, and fasting blood glucose levels were determined using an A5800 automated chemistry analyzer (Brea). Subjects were classified as euthyroid based on these laboratory parameters. The reference ranges were as follows: TSH (0.34–5.60 μIU/mL), FT3 (3.09–9.70 pmol/L), and FT4 (7.64–16.03 pmol/L).

### 2.7. Statistical analysis

Study data were entered using the Epidata 3.1 software, and analyses were performed with SPSS 22.0 software.^[16]^ Pearson’s chi-square test was used to compare proportions between groups. For measurement data meeting the assumptions of normality and homogeneity of variance, statistical differences between groups were assessed using either one-way analysis of variance or an independent samples *t* test. When these assumptions were not met, the Kruskal–Wallis rank sum test was applied.^[17]^ Unconditional logistic regression analysis was performed to adjust for confounding variables such as age, sex, BMI, smoking history, and drinking history, in order to determine the independent association between thyroid hormone levels and MetS as well as CAS. Spearman’s correlation test was used to examine relationships among parameters, including FT3, TG, BMI, SBP, and Gensini score in patients with CAS. Multivariate linear regression analysis was adjusted for confounders such as age, BMI, SBP, DBP, and glucose levels to explore the linear relationship between thyroid hormone levels and the Gensini score. GraphPad Prism 9.0 software was used for illustration.

## 3. Results

### 3.1. Intergroup comparative analysis

#### 3.1.1. Metabolic syndrome

A total of 947 participants were enrolled in this study, with 798 in the non-MetS group (84.27%) and 149 in the MetS group (15.73%). The MetS group had higher proportions of males, individuals with a history of smoking, and those with a history of drinking compared with the non-MetS group (*P* = .001, *P* = .002, and *P* < .001, respectively). In contrast to the non-MetS group, the MetS group showed increased SBP, DBP, TG, glucose, BMI, and Gensini score, along with a significant decrease in HDL levels (all *P* < .001). There was no statistically significant difference in age between the 2 groups (*P* > .05). Further details are presented in Table 1.

**Table 1:** Intergroup analysis of metabolic and thyroid function indicators, Gensini score, and other clinical parameters between MetS and non-MetS groups.

Variables	Non-MetS (n = 798)	MetS (n = 149)	*P*
Age (yr)	62.00 (54.00–69.00)	62.00 (54.00–68.00)	.352
Systolic blood pressure (mm Hg)	121.50 (110.00–135.75)	141.00 (128.00–152.00)	**<.001**
Diastolic blood pressure (mm Hg)	75.00 (68.00–82.75)	86.00 (77.00–92.00)	**<.001**
Triglyceride (mmol/L)	1.26 (0.94–1.71)	2.03 (1.67–2.64)	**<.001**
High-density lipoprotein (mmol/L)	1.14 (0.98–1.34)	0.95 (0.84–1.10)	**<.001**
Glucose (mmol/L)	4.94 (4.48–5.39)	5.83 (5.00–7.52)	**<.001**
Thyroid-stimulating hormone (μIU/mL)	1.95 (1.33–2.86)	2.08 (1.36–2.68)	.915
Total triiodothyronine (nmol/L)	1.35 (1.16–1.52)	1.37 (1.21–1.55)	.168
Total tetraiodothyronine (nmol/L)	107.35 (93.40–120.40)	106.52 (94.94–118.55)	.572
Free triiodothyronine (pmmol/L)	5.01 (4.57–5.50)	5.18 (4.85–5.59)	**.002**
Free tetraiodothyronine (pmmol/L)	11.13 (9.98–12.35)	10.83 (9.81–12.13)	.122
Gensini score	4.00 (0.00–14.00)	8.50 (0.00–32.00)	**<.001**
Body mass index (kg/m^2^)	23.06 (21.27–24.80)	26.57 (25.47–28.69)	**<.001**
FT3/FT4	0.45 (0.40–0.51)	0.47 (0.41–0.55)	**.009**
Gender, n (%)			**.001**
Female	375 (46.99)	49 (32.89)	
Male	423 (53.01)	100 (67.11)	
Smoking, n (%)			**.002**
No	559 (70.05)	85 (57.05)	
Yes	239 (29.95)	64 (42.95)	
Drinking, n (%)			**<.001**
No	612 (76.69)	95 (63.76)	
Yes	186 (23.31)	54 (36.24)	

Bold values indicate statistically significant differences (*P* < .05).

FT3/FT4 = free triiodothyronine/free thyroxine ratio, MetS = metabolic syndrome.

**Figure 1.**
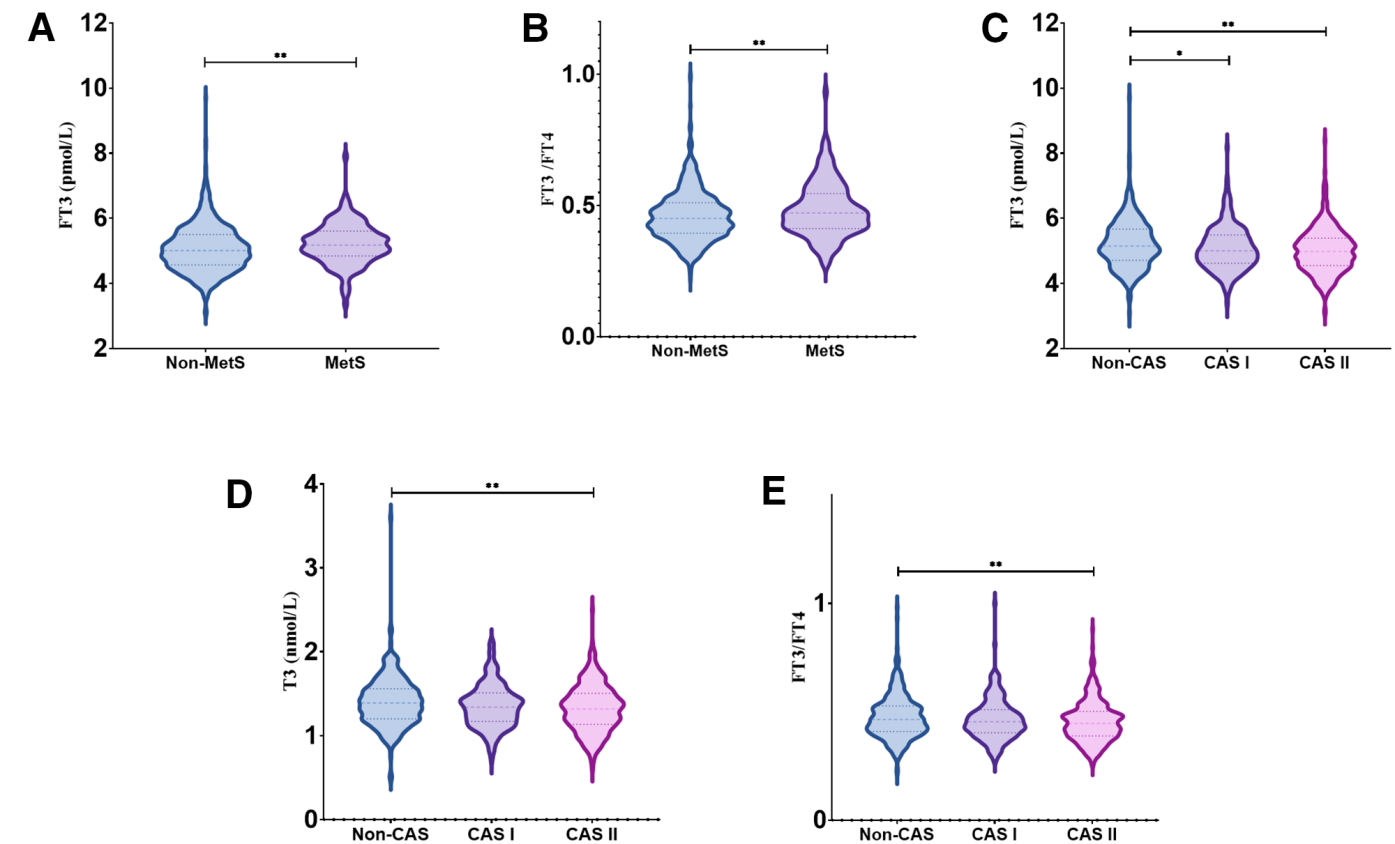
(A) Comparison of FT3 levels between the MetS and non-MetS groups. (B) Comparison of the FT3/FT4 ratio between the MetS and non-MetS groups. (C) Comparison of FT3 levels among groups stratified by severity of CAS. (D) Comparison of T3 levels among groups stratified by CAS severity. (E) Comparison of the FT3/FT4 ratio among groups stratified by CAS severity. *P < .05, **P < .01. CAS = coronary artery stenosis, FT3 = free triiodothyronine, FT3/FT4 = free triiodothyronine/free thyroxine ratio, FT4 = free thyroxine, MetS = metabolic syndrome, T3 = total triiodothyronine.

The intergroup comparison of thyroid function indicators showed that FT3 and the FT3/FT4 ratio were significantly higher in the MetS group than in the non-MetS group (*P* = .002, *P* = .009, all *P* < .05; Fig. 1A, B). However, no significant differences were observed in TSH, T3, T4, or FT4 between the 2 groups (all *P* > .05). After adjusting for confounding factors including sex, age, smoking history, drinking history, and CAS using multivariate logistic regression, the significant differences in FT3 and FT3/FT4 between the groups remained (odds ratio [OR] [95% CI] = 1.341 [1.049–1.713], *P* = .019; OR [95% CI] = 12.888 [2.245–73.998], *P* = .004, all *P* < .05).

### 3.2. Intergroup analysis

#### 3.2.1. Coronary artery stenosis

Herein, there were 331 cases in the non-CAS group (34.95%), 227 cases in the CAS I group (23.97%), and 389 cases in the CAS II group (41.08%). Significant differences were observed among the groups in terms of sex, smoking history, drinking history, and MetS (*P* < .001, *P* < .001, *P* = .005, and *P* = .005, respectively). Compared with the non-CAS group, the CAS I group had higher levels of age, SBP, and Gensini score (*P* < .001, *P* = .041, and *P* < .001, all *P* < .05). In contrast to the non-CAS group, the CAS II group showed higher age, SBP, Gensini score, and glucose, along with a decrease in HDL levels (all *P* < .001). Further details are presented in Table 2.

**Table 2:** Intergroup differences in metabolic and thyroid function parameters and other clinical variables by coronary stenosis severity groups.

Variables	Non-CAS (n = 331)	CAS I (n = 227)	CAS II (n = 389)	*P*
Age (yr)	57.00 (51.00–64.50)	63.00 (56.00–69.00)*	64.00 (58.00–71.00)*	**<.001**
Systolic blood pressure (mm Hg)	120.00 (108.00–135.00)	124.00 (111.50–140.00)*	128.00 (116.00–142.00)*^,^#	**<.001**
Diastolic blood pressure (mm Hg)	77.00 (70.00–84.50)	75.00 (70.00–84.00)	77.00 (69.00–85.00)	.723
Triglyceride (mmol/L)	1.35 (0.98–1.94)	1.32 (0.96–1.82)	1.38 (1.03–1.97)	.149
High-density lipoprotein (mmol/L)	1.15 (0.98–1.35)△	1.15 (0.97–1.35)△	1.05 (0.91–1.24)	**<.001**
Glucose (mmol/L)	4.93 (4.45–5.37)	5.00 (4.54–5.39)	5.05 (4.57–5.98)*^,^#	**<.001**
Thyroid-stimulating hormone (μIU/mL)	1.99 (1.36–2.75)	1.94 (1.33–2.81)	1.97 (1.29–2.90)	.959
Total triiodothyronine (nmol/L)	1.39 (1.20–1.56)	1.34 (1.17–1.50)	1.32 (1.14–1.50)*	**.004**
Total tetraiodothyronine (nmol/L)	108.01 (93.84–119.77)	109.58 (93.91–121.74)	105.40 (92.56–119.08)	.322
Free triiodothyronine (pmmol/L)	5.15 (4.72–5.67)#	5.00 (4.62–5.49)	4.98 (4.55–5.39)*	**<.001**
Free tetraiodothyronine (pmmol/L)	11.06 (10.00–12.34)	11.01 (9.89–12.07)	11.08 (9.93–12.36)	.925
Gensini score	0.00 (0.00–0.00)	5.00 (2.50–6.50)*	21.50 (11.00–40.00)*^,^#	**<.001**
Body mass index (kg/m^2^)	23.31 (21.32–25.41)	23.73 (21.64–25.77)	23.88 (22.03–25.81)	.096
FT3/FT4	0.46 (0.41–0.53)△	0.45 (0.40–0.51)	0.45 (0.39–0.50)	**.024**
Gender, n (%)				**<.001**
Female	193 (58.31)	119 (52.42)	112 (28.79)	
Male	138 (41.69)	108 (47.58)	277 (71.21)	
Smoking, n (%)				**<.001**
No	252 (76.13)	168 (74.01)	224 (57.58)	
Yes	79 (23.87)	59 (25.99)	165 (42.42)	
Drinking, n (%)				**.005**
No	261 (78.85)	177 (77.97)	269 (69.15)	
Yes	70 (21.15)	50 (22.03)	120 (30.85)	
MetS, n (%)				**.005**
No	290 (87.61)	198 (87.22)	310 (79.69)	
Yes	41 (12.39)	29 (12.78)	79 (20.31)	

Bold values indicate statistically significant differences (*P* < .05).

FT3/FT4 = free triiodothyronine/free thyroxine ratio, MetS = metabolic syndrome.

* *P* < .05 versus non-CAS group.

# *P* < .05 versus CAS I group.

△ *P* < .05 versus CAS II group.

The intergroup comparison of thyroid function indicators showed that the FT3 level in the CAS I group was lower than in the non-CAS group (*P* = .029). The CAS II group exhibited lower levels of FT3, T3, and the FT3/FT4 ratio compared to the non-CAS group (*P* < .001, *P* = .006, and *P* = .001, respectively; Fig. 1C–E). Multivariate logistic regression analysis, controlling for confounding factors such as sex, age, smoking history, drinking history, and MetS, indicated that significant differences in FT3 and T3 persisted between the non-CAS and CAS II groups (OR [95% CI] = 0.747 [0.582–0.959], *P* = .022; OR [95% CI] = 0.481 [0.261–0.885], *P* = .019). However, no statistically significant difference in FT3 was found between the non-CAS and CAS I groups, and no significant difference in the FT3/FT4 ratio was observed among the groups after multivariate logistic regression analysis (*P* > .05).

### 3.3. Correlation analysis

**Figure 2.**
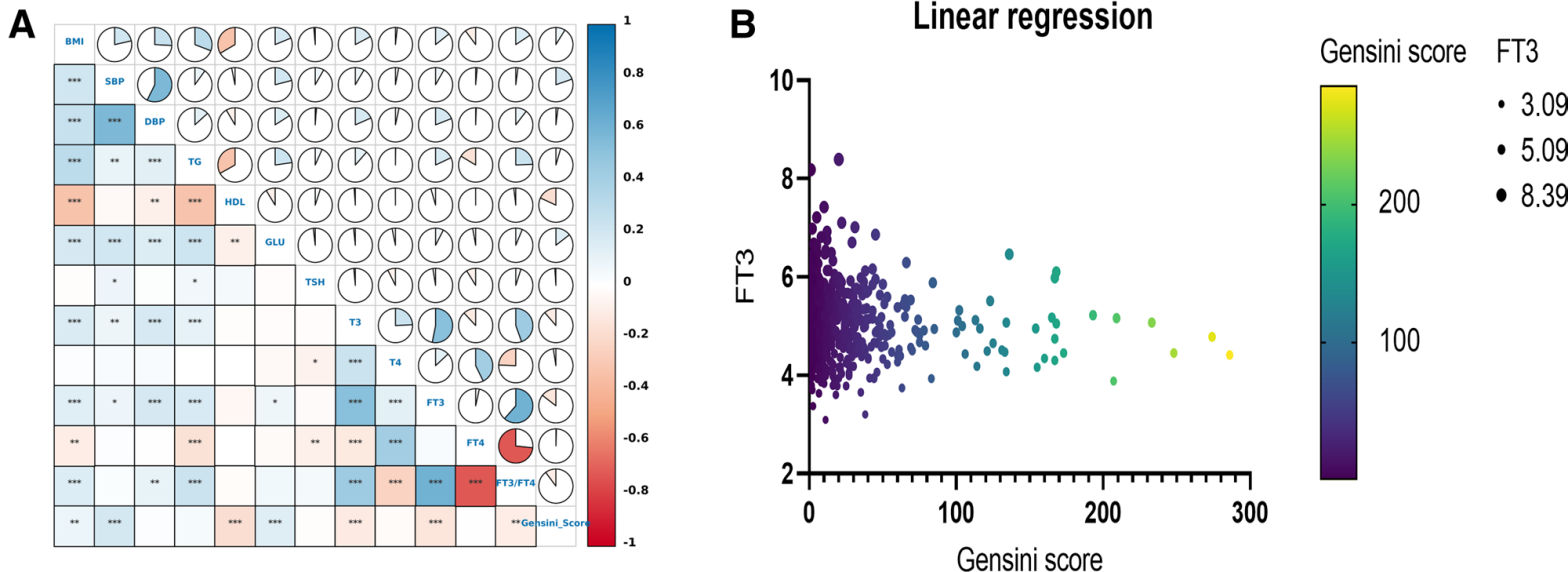
(A) Correlation analysis heatmap of thyroid function parameters, metabolic parameters, and Gensini score. (B) Linear association between FT3 and Gensini score. BMI = body mass index, DBP = diastolic blood pressure, FT3 = free triiodothyronine, FT3/FT4 = free triiodothyronine/free thyroxine ratio, FT4 = free thyroxine, HDL = high-density lipoprotein, SBP = systolic blood pressure, T3 = total triiodothyronine, T4 = total tetraiodothyronine, TG = triglyceride, TSH = thyroid-stimulating hormone.

FT3 was positively correlated with BMI, SBP, DBP, TG, and glucose, and negatively correlated with the Gensini score (ρ = 0.14, 0.08, 0.19, 0.18, 0.08, and −0.15, respectively; all *P* < .05; Fig. 2A). T3 showed positive correlations with BMI, SBP, DBP, and TG, and a negative correlation with the Gensini score (ρ = 0.17, 0.08, 0.18, 0.11, and −0.11, respectively; all *P* < .05; Fig. 2A). The FT3/FT4 ratio was positively correlated with BMI, DBP, and TG, and negatively correlated with the Gensini score (ρ = 0.16, 0.10, 0.24, and −0.10, respectively; all *P* < .05; Fig. 2A). The Gensini score was positively correlated with BMI, SBP, and glucose (ρ = 0.09, 0.19, and 0.15; all *P* < .05), and negatively correlated with HDL, FT3, and FT3/FT4 (ρ = −0.18, −0.15, and −0.10; all *P* < .05; Fig. 2A). Further details are shown in Table 3.

**Table 3:** Correlation analysis of thyroid function parameters, metabolic parameters, and Gensini score.

	BMI	SBP	DBP	TG	HDL	GLU	Gensini score
TSH	−0.01	0.08^*^	0.01	0.07^*^	0.05	−0.02	−0.01
T3	0.17^**^	0.08^**^	0.18^**^	0.11^**^	−0.01	−0.02	−0.11^**^
T4	0.02	0.03	0.03	0.00	−0.00	−0.03	−0.02
FT3	0.14^**^	0.08^*^	0.19^**^	0.18^**^	−0.04	0.08^*^	−0.15^**^
FT4	−0.10^**^	0.01	0.01	−0.16^**^	0.00	−0.03	0.01
FT3/FT4	0.16^**^	0.02	0.10^**^	0.24^**^	−0.01	0.06	−0.10^**^
Gensini score	0.09^**^	0.19^**^	0.02	0.05	−0.18^**^	0.15^**^	1.00

BMI = body mass index, DBP = diastolic blood pressure, FT3 = free triiodothyronine, FT3/FT4 = free triiodothyronine/free thyroxine ratio, FT4 = free thyroxine, HDL = high-density lipoprotein, SBP = systolic blood pressure, T3 = total triiodothyronine, T4 = total tetraiodothyronine, TG = triglyceride, TSH = thyroid-stimulating hormone.

* *P* < .05.

** *P* < .01.

### 3.4. Linear regression analysis

**Figure 3.**
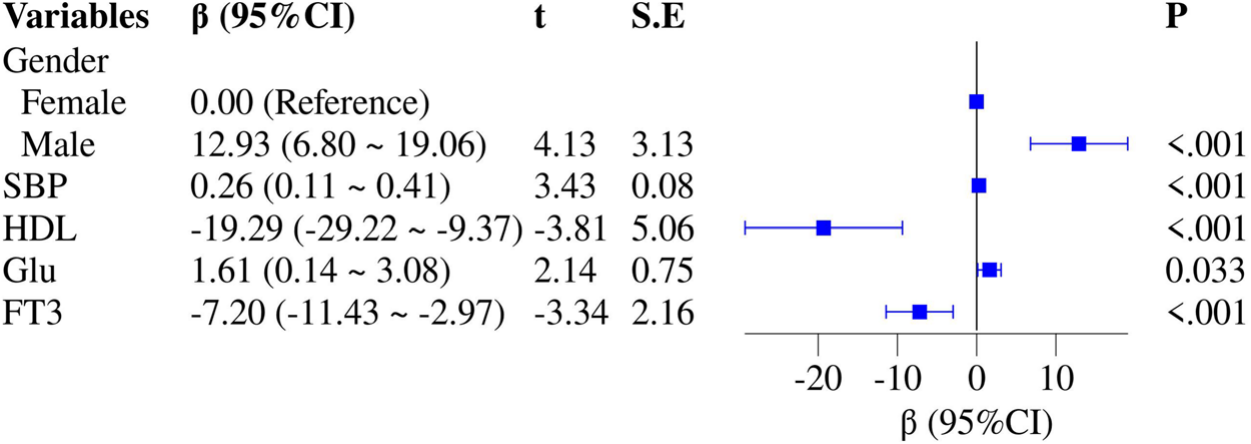
Multivariate linear regression analysis of the Gensini score with thyroid function and other variables. CI = confidence interval, FT3 = free triiodothyronine, HDL = high-density lipoprotein, SBP = systolic blood pressure.

The univariate linear regression analysis showed that the Gensini score was negatively and linearly correlated with FT3 and HDL, and positively correlated with sex, smoking, BMI, SBP, and glucose (*P* = .027, *P* < .001, *P* < .001, *P* < .001, *P* = .016, *P* = .001, and *P* = .004, respectively; Fig. 2B). Further multivariate linear regression analysis revealed that FT3 retained a significant linear negative association with the Gensini score (β [95% CI] = −7.20 [−11.43 to −2.97], *P* < .001; Fig. 3).

## 4. Discussion

The present study focused on individuals with normal thyroid function and conducted an in-depth examination of the relationship between thyroid hormones, MetS, and CAS. The findings indicated that FT3 was positively correlated with MetS and negatively correlated with the severity of coronary stenosis. This complex pattern of correlations reflects the multifaceted mechanisms underlying thyroid hormone action.^[18,19]^

The findings indicate a positive correlation between FT3 and BMI, blood pressure, TGs, and blood glucose levels. As the biologically active form of thyroid hormone, FT3 plays a key role in energy metabolism by enhancing energy expenditure through activation of β-adrenergic receptors and mitochondrial uncoupling proteins, thereby contributing to the maintenance of energy homeostasis.^[20-22]^ However, this dynamic becomes more complex in the context of insulin resistance. Numerous studies have demonstrated a positive correlation between FT3 levels and the insulin resistance index across various states of glucose metabolism.^[23]^ Insulin resistance impairs cellular glucose uptake and utilization. Consequently, although FT3 promotes lipolysis and energy release, it does not effectively improve glucose metabolism and may worsen metabolic dysfunction.^[24,25]^ Concurrently, in vitro studies suggest that FT3 may enhance TG synthesis and accumulation by modulating lipid metabolism-related transcription factors, such as sterol regulatory element-binding protein-1c, thereby contributing to the development of MetS.^[26]^

Notably, an elevated FT3/FT4 ratio is independently associated with the incidence of MetS.^[23]^ Although both FT3 and FT4 are thyroid hormones, they have distinct physiological effects. FT3 is more biologically active, while FT4 primarily serves as a precursor or reserve form.^[27]^ An increased FT3/FT4 ratio may indicate a relative rise in active thyroid hormone levels in vivo, potentially worsening metabolic disturbances.^[28]^ This alteration may reflect an imbalance in thyroid hormone metabolism, thereby influencing the risk of developing MetS. Previous research by Ladislav et al demonstrated a significant correlation between the FT3/FT4 ratio and insulin resistance in individuals with normal thyroid function, supporting the findings of our study.^[23]^

FT3 is positively correlated with MetS but negatively associated with the severity of CAS, a phenomenon that may be primarily attributed to its dual roles as the biologically active form of thyroid hormone, mediating both metabolic activation and cardiovascular protection.^[29-31]^ On the one hand, FT3 enhances overall metabolic activity by activating mitochondrial oxidative phosphorylation and promoting lipolysis and glucose uptake. Elevated FT3 levels and a higher FT3/FT4 ratio may directly induce abnormalities in core components of MetS, such as insulin resistance, hypertriglyceridemia, and nonalcoholic fatty liver disease, thus serving as an independent risk factor for MetS and nonalcoholic fatty liver disease.^[29,32]^ On the other hand, FT3 plays a critical regulatory role in the formation of coronary collateral circulation and the maintenance of vascular endothelial function. Reduced FT3 levels may accelerate the progression of coronary atherosclerosis by inhibiting endothelial nitric oxide production and exacerbating myocardial fibrosis and lipid metabolism disorders. In addition, low FT3 levels may represent a compensatory response to coronary artery injury; patients with low T3 syndrome exhibit substantially higher CAS severity than euthyroid individuals, while low FT3 levels have been associated with an increased risk of CAS. Furthermore, reduced FT3 levels are an independent predictor of impaired coronary collateral circulation in patients with stable coronary heart disease.^[33-35]^ The results of this regression analysis suggest that, after adjusting for confounding factors such as MetS, low FT3 levels are independently associated with severe coronary stenosis (CAS II group), but not with mild coronary stenosis. This may further indicate that low FT3 levels represent a protective response in severe coronary stenosis.

Nevertheless, this study has certain limitations. As a cross-sectional design, it cannot establish causal relationships between FT3, the FT3/FT4 ratio, MetS, and CAS. As this was an exploratory study with few groups, we did not use adjusted *P* values, which may increase the risk of Type I error. Moreover, all participants were enrolled from a single center, which may restrict the generalizability of our findings to some extent. Therefore, future research should consider prospective cohort or interventional study designs to clarify these causal associations more effectively. Importantly, the molecular mechanisms underlying the coronary protective effects of FT3 warrant further in vivo and in vitro validation. Moreover, this study measured only specific thyroid hormone indicators and did not include other relevant markers, such as thyroid antibodies, which could provide a more comprehensive understanding of the relationship between thyroid function and disease. Subsequent research should incorporate these additional markers to explore the complex relationship between thyroid function, MetS, and CAS in greater depth.

## Acknowledgments

This work was supported by the Weifang Science and Technology Development Plan Project (2025YX075) and the Army Clinical Key Cultivation Specialty Construction Funds.

## Author contributions

**Writing – original draft:** Mengran Guo.

**Methodology:** Chao Liu.

**Conceptualization:** Dan Lv.

**Project administration:** Qingshu Lin.

**Investigation:** Ali Zhang.

**Writing – review & editing:** Peng Cai.
